# Completeness of Retention Data and Determinants of Attrition in Birth Cohorts of Very Preterm Infants: A Systematic Review

**DOI:** 10.3389/fped.2021.529733

**Published:** 2021-02-17

**Authors:** Raquel Teixeira, Ana Catarina Queiroga, Ana Isabel Freitas, Elsa Lorthe, Ana Cristina Santos, Carla Moreira, Henrique Barros

**Affiliations:** ^1^EPIUnit – Instituto de Saúde Pública, Universidade do Porto, Porto, Portugal; ^2^Departamento de Ciências da Saúde Pública e Forenses e Educação Médica, Faculdade de Medicina, Universidade do Porto, Porto, Portugal; ^3^CMAT - Centro de Matemática, Universidade do Minho, Braga, Portugal

**Keywords:** cohort studies, infant, premature, very preterm infants, lost to follow-up, retention

## Abstract

**Background:** Birth cohorts provided essential knowledge for clinical and public health decision-making. However, little is known about retention and determinants of attrition in these specific longitudinal studies, although characterizing predictors of attrition sets the path to mitigate its occurrence and to promote valid inferences. We systematically reviewed retention in follow-ups of birth cohorts of very preterm or very low birth weight infants and the determinants of attrition. PROSPERO registration number: CRD42017082672.

**Methods:** Publications were identified through PubMed®, Scopus, Web of Science, and Cochrane Library databases from inception to December 2017. Studies were included when reporting at least one of the following: retention at follow-ups, reasons for attrition, or characteristics of non-participants. Quality assessment was conducted using the completeness of the report of participation features in the articles. Non-participant's characteristics were presented using descriptive statistics. Local polynomial regression was used to describe overall retention trends over years of follow-up.

**Results:** We identified 57 eligible publications, reporting on 39 birth cohorts and describing 83 follow-up evaluations. The overall median retention was 87% (p25–p75:75.8–93.6), ranging from 14.6 to 100%. Overall, retention showed a downward trend with increasing child age. Completeness of retention report was considered “enough” in only 36.8% of publications. Considering the 33 (57.9%) publications providing information on participants and non-participants, and although no formal meta-analysis was performed, it was evident that participants lost to follow-up were more often male, had foreign-born, multiparous, and younger mothers, and with a lower socioeconomic status.

**Conclusion:** This systematic review evidenced a lack of detailed data on retention, which may threaten the potential use of evidence derived from cohort studies of very preterm infants for clinical and public health purpose. It supports the requirement for a standardized presentation of retention features responding to current guidelines.

## Introduction

During the last decades, improvements in obstetric and neonatal care led to an increase in the survival of very preterm (born before 32 complete weeks of gestation) or very low birth weight (<1,500 g) infants (1–3). However, these infants are at higher risk of developing short- and long-term health complications (4–6), the families face a high psychosocial and emotional burden (7, 8), and there is a significant societal impact due to continuing health care and educational needs (9).

Birth cohorts of preterm infants have been established since the late 1970s to quantify clinical outcomes, understand the determinants and consequences of prematurity, and provide data to support health policies. The longitudinal nature of this type of study affords a life-course approach that is crucial to elucidate and improve the long-term effects of being born preterm. However, as cohort studies require periodic contact and examinations, they demand from research participants a strong commitment along the life course and thus are more susceptible to attrition. Attrition occurs when initially recruited subjects fail to maintain their participation in a study following enrollment and hence are considered lost to follow-up (10). Described losses to follow-up on studies of very preterm infants indicate that low socioeconomic position, low maternal educational level, and high frequency of cognitive impairments and behavioral disorders in childhood may be the most likely determinants of attrition (11, 12), but there is still a gap of knowledge on this field.

Attrition can undermine the representativeness of the population of interest, bias the measured associations, and compromise the precision of the estimates, threatening internal and external validity of the study (13, 14). Therefore, improving the recruitment, tracking, and retention of the participants is crucial to guarantee the reliability of longitudinal studies, and several strategies have been discussed to maximize retention and keep the losses to a minimum. Incentives (monetary or other) and alternative methods of data collection have been described as feasible and associated with improved retention in follow-up evaluations (15, 16).

Despite the well-known potential for bias that non-random losses can entail, attrition is a frequently understudied and underreported phenomenon in epidemiological studies (17, 18). Thus, we systematically reviewed publications presenting data on retention in follow-ups of birth cohorts of very preterm or very low birth weight infants or determinants of attrition.

## Methods

We conducted a systematic search of the peer-refereed literature based on the approach recommended by the Preferred Reported Items for Systematic Reviews and Meta-Analyses (PRISMA) Statement (19). The protocol for the review was registered in PROSPERO (Registration ID: CRD42017082672).

### Search Strategy and Study Selection

**Figure 1:**
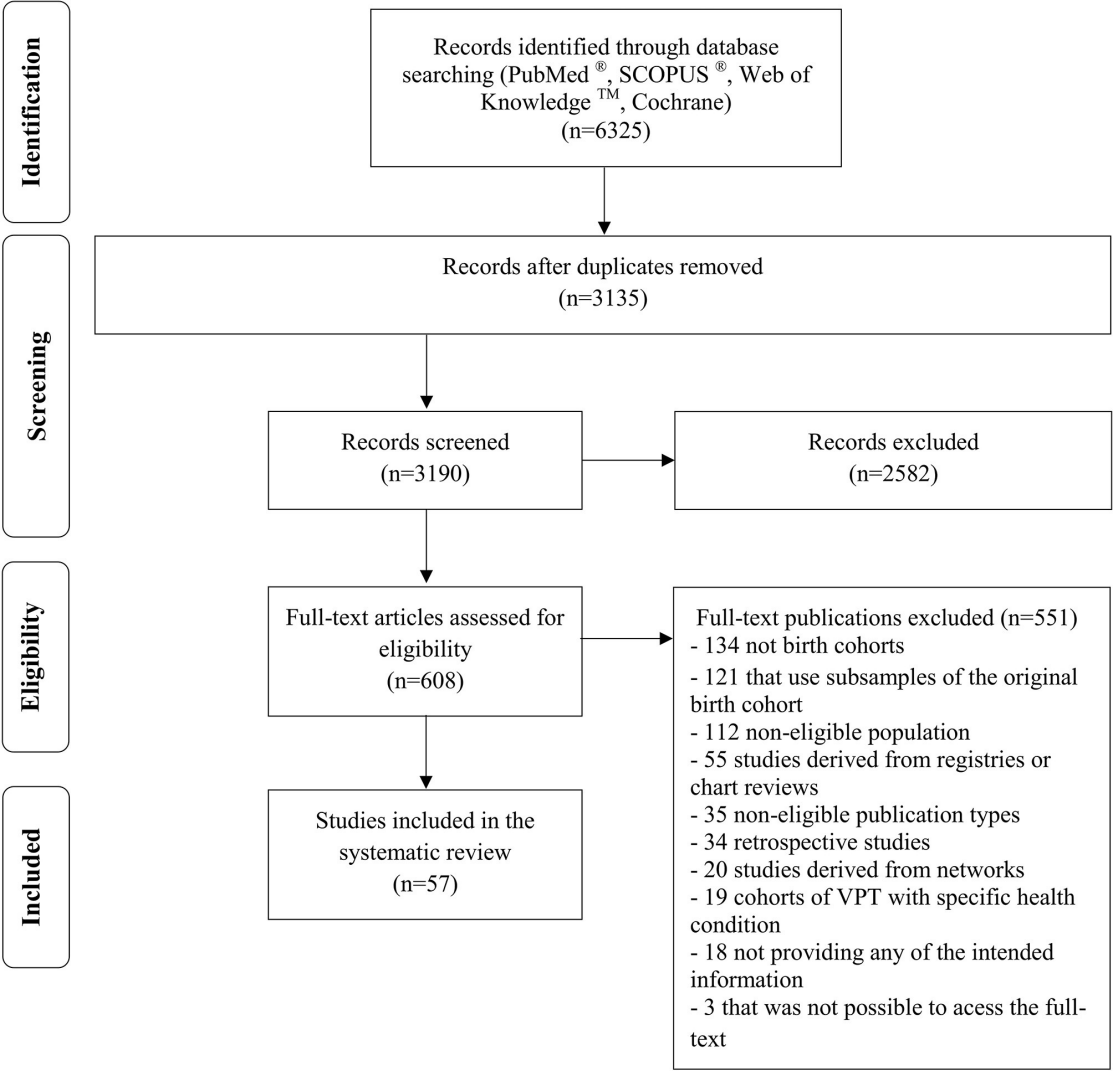
PRISMA systematic review flowchart. Search Expression—PubMed®: (participation OR “response rate” OR “response proportion” OR “follow-up rate” OR “follow-up proportion” OR “follow-up participation” OR “follow-up strategy” OR lost to follow-tip” OR “loss to follow-up” OR “retention rate” OR “retention proportion” OR “non-compliance” OR “non-response” OR “refusal” OR “attrition” OR “dropping out” OR “dropout” OR “drop-out” OR “reimbursement” OR “incentive” OR “motivation”) AND (“cohort” OR “prospective” OR “retrospective” OR “longitudinal” OR observational OR “population-based”) AND (“preterm” OR “premature” OR “prematurity” OR “low birth weight” OR “low birthweight”).

Two search strategies were applied. First, PubMed®, Scopus, Web of Science, and Cochrane electronic databases were searched from inception to December 2017, to identify publications reporting retention in follow-up evaluations of birth cohorts of very preterm or very low birth weight infants. The search expression is provided in the systematic review flowchart shown in Figure 1. Second, reference lists of all publications selected for review were manually searched for additional studies. To clearly classify the relevant publications, we set the following definition of birth cohort: a representative sample of infants born during a particular and prespecified period of time, with enrollment during pregnancy, at birth or in Neonatal Intensive Care Unit, and with at least one follow-up evaluation.

The eligibility criteria were as follows: (a) birth cohort studies; (b) participants born preterm, i.e., <37 weeks of gestational age or low birthweight, i.e., <2,500 g; (c) studies that provided at least one of the following: retention (or data to calculate it), attrition (or data to calculate it), reasons for attrition, and characteristics of non-participants. Letters to the editor, commentaries, case reports, and case series were excluded. Excluded studies also comprise those derived from networks, clinical follow-up programs, birth cohorts of specific health conditions, or those that use subsamples of the original birth cohort. We did not apply any restrictions on the types of exposures or outcomes studied.

To reduce the potential for reviewer bias, titles and abstracts of all identified records were independently screened by at least two authors (RT, ACQ, AIF, and EL) and checked for agreement. Subsequently, the full text of potentially relevant studies was read and independently screened for the eligibility criteria. Discrepancies in the study selection were resolved by consensus or were discussed with a third author (ACS or HB) for a final decision.

We measured interrater agreement to decide on titles and abstract screening using the Cohen's Kappa (20). The obtained coefficient indicated a good agreement between raters (κ = 0.745, *p* < 0.001).

### Data Extraction and Quality Assessment

We defined a search expression focusing on preterm birth and low birth weight to maximize the search scope and thereafter the identification of relevant studies. However, we only extracted data from publications where information on very preterm (<32 weeks) or very low birth weight participants (<1,500 g) was reported. Data were extracted from each publication by two independent reviewers (RT, ACQ, AIF, and EL) and included the following: first author, publication year, country where the study was conducted, recruitment start year, baseline sample size, age at the follow-up (an approximate estimate of the length of follow-up in birth cohorts), retention, attrition, use of retention strategies [incentives (financial or other), reimbursements, reminders, strategies to keep in contact], reasons for attrition, and socioeconomic, demographic, and health-related differences between participants and non-participants.

Retention was defined as the number of participants retained at follow-up divided by the number of eligible participants and presented as percentage. We only extracted retention or attrition data from the content of the included publications, as such none of the references cited in the methods section of the included publications were investigated.

When two or more different publications described the same birth cohort and same follow-up evaluation, but reported different retention proportions, we selected the publication that reported the higher sample of eligible participants and/or the higher follow-up retention rate and excluded the others. This decision was taken to assure we were not selecting subsample studies. Such discrepancies in retention between publications might happen due to different eligibility criteria or different outcome assessed.

The completeness of the report of retention features was classified into one of three ordinal categories [adapted from Morton et al. (21): (1) “scarce information” (when only the retention proportion was reported or necessary data to calculate it); (2) “some information” (when studies provided retention plus data on the characteristics of non-participants or gave information about the reasons for attrition); or (3) “enough information” (retention and both data on the characteristics of non-participants and information about the reasons for attrition).

We hypothesized that the publication of the Strengthening the Reporting of Observational studies in Epidemiology (STROBE) Statement (22) in 2007 could influence the degree of completeness of retention features in the publications reporting on the relevant cohorts, and therefore, completeness was compared by categories of publication year (≤2007, 2008–2010, ≥2011). The first 3 years after the STROBE Statement was included in an intermediate category to take into account the time needed to implement the guideline.

### Data Synthesis and Analysis

General characteristics of the publications and birth cohorts were presented as counts and proportions for categorical variables. For continuous variables, we reported mean and standard deviation or median and percentile 25 and percentile 75 (p25–p75). Continuous variables were compared using Student's t, Mann–Whitney, or ANOVA tests, and the categorical ones were compared using a chi-square test, as appropriate. The strength of associations was measured using Pearson or Spearman coefficients of correlation, as appropriate. Our search strategy did not collect all follow-up evaluations performed by each birth cohort; thus, correlations and mean comparisons were performed using the retention in the earliest follow-up evaluation extracted by each birth cohort.

We performed mixed-effects models using a local polynomial regression between years of follow-up and retention proportion in each follow-up evaluation (time points), from all birth cohorts (random effects). Since the data are unbalanced, we applied this smoothing method in order to capture the potential relationship between those variables through a more flexible approach, allowing the data points themselves to determine the form of the fitted curve. The generalized cross-validation method was used to select the optimal bandwidth taking advantage of the procedure called “leaving-one-out,” where each observation is successively “left out” from the analysis in order to perform the optimal fit (23–25). This analysis was conducted using the fANCOVA and lme4 R package.

Reasons for attrition were listed as provided in the original papers and quantified as counts and proportions. The differences between participants and non-participants were examined taking into account the direction of the associations and the statistical significance.

Data analysis was performed using R® statistical software version 3.0.1 (2013) and the Statistical Package for Social Science for Windows (SPSS) version 23.0.0 (2015).

## Results

### Publications Characteristics

The combined search strategy returned 6325 publications. After removing duplicates and title and abstract screening, 608 publications remained for full-text assessment. We removed 551 publications based on full-text evaluation, leaving 57 publications based on the inclusion criteria (Figure 1). These 57 publications corresponded to 39 birth cohorts, providing information for 83 follow-ups.

Thirty-two publications were from Europe [five from France (26–30), five from Sweden (31–35), five from Norway (36–40), three from The Netherlands (41–43), three from the United Kingdom (11, 44, 45), two from Denmark (46, 47), two from Germany (48, 49), two from Italy (50, 51), two from Finland (52, 53), one from Scotland (54), one from Austria (55), and one from Belgium (56)], seven from North America [five from Canada (57–61), two from the United States of America (62, 63)], 17 from Oceania [16 from Australia (64–79) and one from New Zealand (80)], and one from Asia [Taiwan (81)].

The completeness of retention report was considered as “scarce” in 5 publications (8.8%), “some information” in 31 (54.4%), and “enough information” in 21 (36.8%) (Table 1). Thirty-one articles (54.4%) were published after the STROBE initiative. However, there were no clear trends in the proportion of publications assessed as “enough information” by publication year [(≤2007), 34.6% vs. (2008–2010), 54.5% vs. (≥2011), 30%, *p* = 0.44].

**Table 1:** Overall characteristics of (a) publications included and (b) birth cohorts identified in the systematic review.

	**(a) Publications (*n* = 57)**	**(b) Birth cohorts (*n* = 39)**
**Region (N, %)**		
Europe	32 (56.1)	27 (69.2)
Oceania	17 (29.8)	8 (20.5)
North America	7 (12.3)	3 (7.7)
Asia	1 (1.8)	1 (2.6)
**Year of publication (N, %)**		
≤2007	26 (45.6)	Not applicable
2008–2010 STROBE^a^	11 (19.3)	Not applicable
≥2011 STROBE^a^	20 (35.1)	Not applicable
**Participants' age at follow-up (N, %)**		
≤2 years	17 (29.8)	Not applicable
3–5 years	13 (22.8)	Not applicable
>5 years	27 (47.4)	Not applicable
**Completeness (N, %)**		
“Scarce information”	5 (8.8)	Not applicable
“Some information”	31 (54.4)	Not applicable
“Enough information”	21 (36.8)	
**Recruitment start year (median, p25–p75)**	Not applicable	1994 (1985–2001)
**Baseline sample size (median, p25–p75)**	Not applicable	397 (250–707)
**Recruitment setting (N, %)**		
Single center	17 (29.8)	13 (33.3)
Multicenter	40 (70.2)	26 (66.7)

*STROBE, Strengthening the Reporting of Observational Studies in Epidemiology*.

a *After STROBE Statement*.

### Birth Cohort's Characteristics and Retention Features

The initial year of recruitment ranged from 1977 to 2011 (Table 2) with 27 birth cohorts (67.5%) having a multicenter recruitment (Table 1). The baseline sample size varied considerably, with a median of 405 participants (p25–p75: 254–707), ranging from 47 in a Norwegian cohort (39) to 3,964 in the French EPIPAGE 2 cohort (26).

**Table 2:** Identified birth cohorts and retention by follow-up evaluation.

**Birth cohorts of very preterm infants**	**Recruitment start year**	**y1**	**y2**	**y3**	**y4**	**y5**	**y6**	**y7**	**y8**	**y9**	**y10**	**y11**	**y12**	**y13**	**y14**	**y15**	**y16**	**y17**	**y18**	**y19**	**y20**	**y21**	**y22**	**y23**	**y24**	**y32**
McMaster's Neonatal Follow-up Program	1977								90						91									90		60.6
HeSva–Helsinki Study of Very Low Birth Weight Adults	1978																						65.1			
Victorian Infant Collaborative Study Group, 1979	1979		93.3			96			93.3						90											
Dinessen, S.J.	1980		99.1		89.5														95.8							
Rogers, M.	1981																	67								
POPS—Project on Preterm and Small for Gestational Age	1983		97.4			96				84.4	71.5				88.7					74						
Hall, A.	1984								95.6																	
BLS—Bavarian Longitudinal Study	1985						84		88					76												
Gross S.	1985										94															
Victorian Infant Collaborative Study Group, 1985	1985		99.5			98.6																				
Horwood L.J.	1986							91.4																		
VLBW follow-up 1986–1988	1986														70					72				67		
Finnstrom O. 1987	1987									81			86													
“1000 g” National Swedish cohort	1990											97														
Finnstrom O. 1990	1990			98.1																						
Vederhus, B.	1991										100															
Victorian Infant Collaborative Study Group, 1991	1991		95.6			94			92.3									76.5								
Danks M.	1992												45.7													
Bertino E.	1994		84.7																							
ETFOL	1994					94																				
The EPICure Study	1995		92				78					71														
Finnish ELBW Cohort Study	1996					94																				
Leiden Follow-up Project on Prematurity	1996	71	64																							
Brevaut-Malaty V.	1997						71.4		89																	
EPIPAGE—Étude épidémiologique sur les petits âges gestationnels	1997		83			77			61																	
Victorian Infant Collaborative Study Group, 1997	1997	.							94																	
EPIBEL—Extremely Preterm Infants in Belgium study	1999			84																						
PEP—Project Extreme Prematurity, 1999–2000	1999					82.3																				
Tu Y.	2001					85.8																				
Ullman, H.	2001		98.2			87.1		88.4																		
VIBeS—Victorian Infant Brain Studies	2001		97			89	88																			
ACTION follow-up project	2003		83																							
Kiechl-Kohlendorfer U.	2003	82																								
The Newborn Lung Project, 2003	2003		73.4																							
EXPRESS—Extremely Preterm Infants in Sweden Study	2004		84.5				93.9																			
Lower Saxony Longitudinal Study of Prematurity	2004		73.5			63.5					14.6															
Victorian Infant Collaborative Study Group 1, 2005	2005		95						87																	
The Epicure Study 2	2006			55.3																						
EPIPAGE 2—Étude épidémiologique sur les petits âges gestationnels	2011		83.4																							

**Figure 2:**
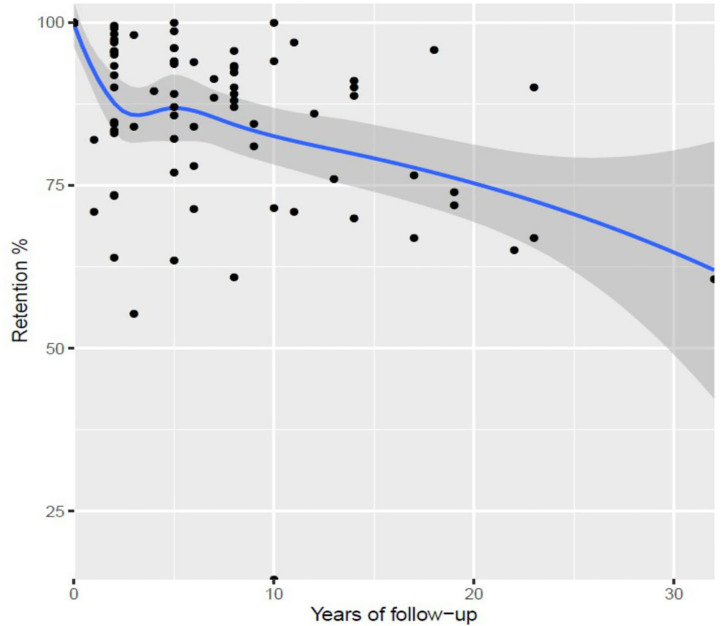
Best fit for retention along with follow-ups, all births cohorts (local polynomial regression), with 95% confidence bands.

Considering the 83 follow-up evaluations identified, the overall median of retention was 87% (p25–p75: 75.8–93.6), ranging from 14.6% (49) to 100% (39). Twenty-seven publications (47.4%) reported follow-ups when participants where older than 5 years. No differences were found in retention by categories of age at follow-up: ≤2 years (87.2%; 95% CI, 81.7–92.8), 3–5 years (86.2%; 95% CI, 81.2–91.2), or >5 years (80.9%; 95% CI, 76.1–85.7) (*p* = 0.16). However, the fitting of a local polynomial regression revealed a decline of retention during the first 2 years of follow-up, with a slight recovery up to the 5 years, to consistently decline again during the upcoming years (Figure 2).

No significant correlations were found between retention at first follow-up and year of recruitment (ρ = −0.13, *p* = 0.41) or baseline sample size (ρ = −0.2, *p* = 0.23). In addition, no clear differences were observed in mean proportion of retention between settings of recruitment [single 80.5% (95% CI, 70.4–90.7) vs. multicenter 88.2% (95% CI, 84.1–92.9), *p* = 0.08] or geographical regions [Europe 84.4% (95% CI, 79.6–89.1) vs. North America 86.2% (95% CI, 58.3–100) vs. Oceania 89.6% (95% CI, 74.6–100)], *p* = 0.62.

Any reference to retention strategies was present in three (5.2%) publications: one declaring “no incentives” (51), one declaring “financial incentives” (37), and another referring “other types of strategies” (26) such as offering test results, sending letters or newsletters, and keeping websites.

The reasons for dropping out were described in 36 (63.2%) publications, providing information on 46 follow-ups. The more frequently reported were refusal (*n* = 31, 86.1%), moving abroad (*n* = 14, 40%), and living too far (*n* = 4, 11%). Other reasons were referred in 20 follow-ups using such expressions as “not traceable,” “not possible to contact,” “lost,” and “the child was adopted.”

**Figure 3:**
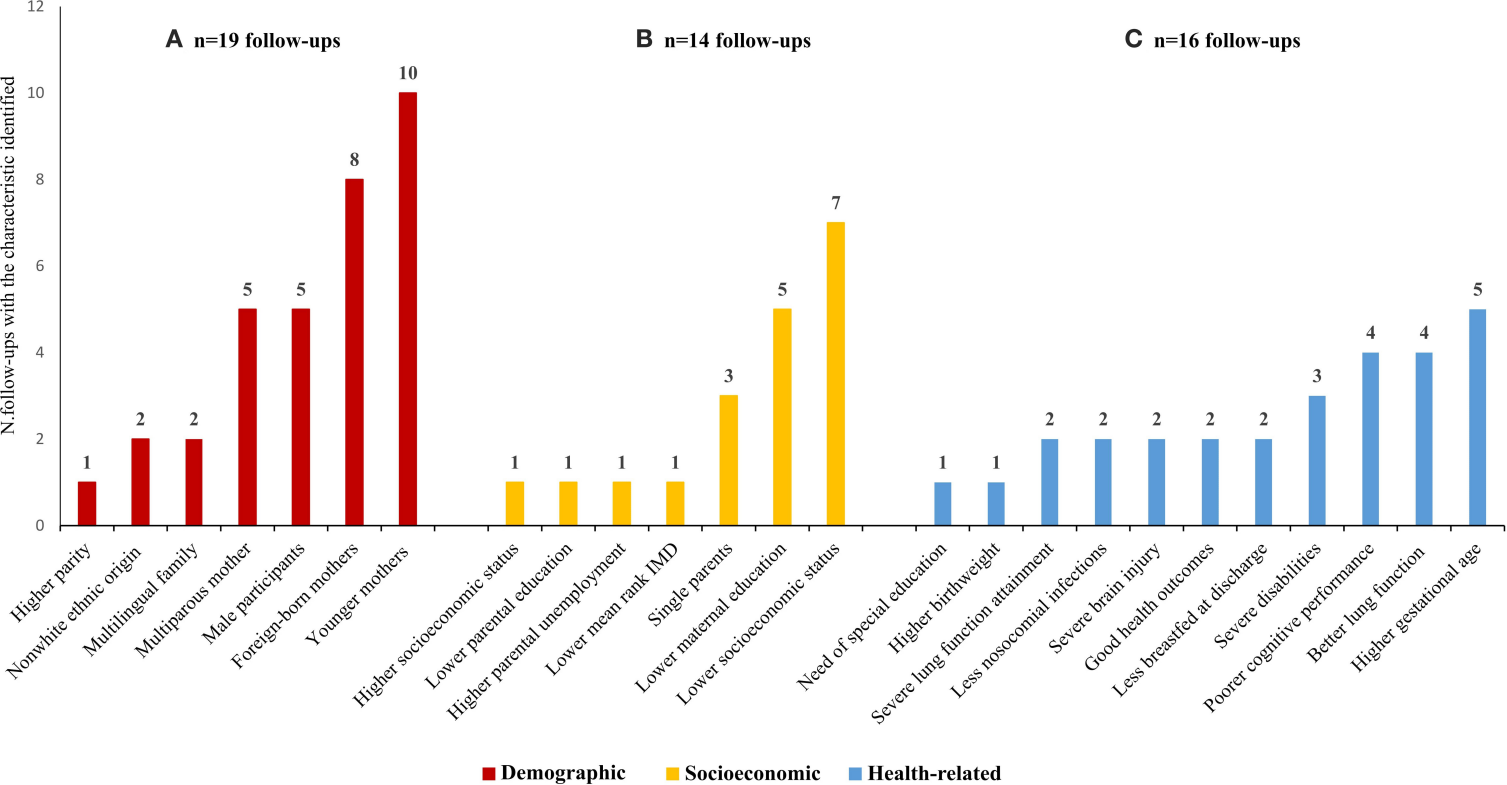
Demographic **(A)**, socieconomic **(B)**, and health-related characteristics **(C)** of non-participants significantly different from participants as assessed in the 40 follow-up evaluations that reported these comparisons. IMD, index of mulitiple deprivation. *Please note that one follow-up often reported more than one characteristic.

The comparison of participants and non-participants was provided by 33 (57.9%) publications, describing 40 follow-ups. Demographic characteristics were investigated in 28 follow-up evaluations (28/40; 70%), and differences were found in 19 (19/28; 67.9%). Non-participants tend to be more frequently male, born to multiparous, foreign-born, and younger mothers. Socioeconomic characteristics were analyzed in 23 (23/40; 57.5%) follow-ups, and differences were found in 14 (14/23; 60.9%). Attrition was more frequent among participants born to mothers with lower educational and lower socioeconomic status. Health-related characteristics were analyzed in 39 (39/40; 97.5%) follow-up evaluations, and differences were identified in 16 (16/39; 41.0%). Non-participants tend to present either the better (higher gestational age, lower frequency of bronchopulmonary dysplasia, chronic lung disease, or nosocomial infections) or the worse health conditions (poorer cognitive performance, higher frequency of brain injury, and overall disabilities) (Figure 3).

## Discussion

This systematic review stresses a relevant lack of data and an inconsistent report of retention and its determinants. It suggests major implications to how findings from cohort studies are interpreted, which may lead to inappropriate decision-making in clinical setting. We identified 57 publications, corresponding to 39 birth cohorts that reported information on 83 follow-up evaluations with a median overall retention proportion of 87%. None of the baseline characteristics tested could explain the wide range of retention (14.6–100%) observed in the reviewed publications. In addition, no difference in retention proportion was found between categories of length of follow-up, although we could detect a downward trend during the first 2 years of child age and after 5 years. In general, we found that the publications lacked detailed information on features associated with retention; nearly two-thirds did not provide information on retention features, i.e., reasons for attrition and characteristics of non-participants.

The publications reviewed presented highly diverse birth cohorts, considering sample size, geographical region, year of recruitment, or ages at the follow-up. Since the abovementioned characteristics could not explain the wide range of retention identified, we expect that other features may have affected retention, namely, data collection methods (82). However, as this information was not consistently reported in the publications, we could not test its effect. Similarly, retention strategies were often not explicitly reported, making impossible to assess if retention was eventually influenced by any such strategies. Incentives, financial and others, have been reported to increase retention (15). Therefore, revealing the use of such strategies in a standardized way would be relevant to understand their potential contribution to mitigate attrition.

The observed downward trend in cohort retention during the first 3 years of follow-up must be interpreted cautiously since this decline was mainly due to one observation with low retention (11). Likewise, the lowest point around 10 years of follow-up was driven by the lowest retention proportion reported among all birth cohorts (49), which is explained by the ongoing status of the follow-up evaluation at the time of the article's publication. Still, the loss of participants with increasing child age can be clearly observed (Figure 2), indicating that the analysis over time may be impacted by the retention. This finding may be linked with the burden on participants and their families due to cumulative contacts, requests, and evaluations (83). Besides, our results pointed out that the most reported reason for attrition were secondary refusals, i.e., after initial agreement to participate. In addition, categories indicating difficulties to track participants (e.g., “not traceable,” “not possible to contact”) were found to have a considerable contribution to the overall reasons for attrition. Qualitative studies about the personal motivation for keeping participating and perceived effect of retention strategies may help to fill the gap of knowledge and further understand this trend.

Conceptually, losses to follow-up can be framed into three categories: missing completely at random (MCAR), in which the probability for a data point to be missing is completely random; missing at random (MAR), in which the probability for a data point to be missing is not related to the missing data, but it is related to some of the observed data; and missing not at random (MNAR), in which the probability of being missing varies for reasons that are unknown to the investigator (unobserved information or to the outcome variable). However, in practice, it is impossible to identify when loss to follow-up is related to unmeasured variables. In a simulation study investigating the statistical effects of attrition in cohort studies (84), the authors advised investigators to assume that loss to follow-up is MNAR and to try to achieve the maximum follow-up rate possible. In this systematic review, it was possible to verify that attrition in birth cohorts of very preterm infants tends to be selective. However, it is important to emphasize that our findings are limited by the lack of relevant information in about 40% of the included publications. The included publications reported differences regarding socioeconomic, demographic, and health-related characteristics. In general, where this information was reported, attrition was related to less advantage social conditions of the parents, such as lower maternal education and lower socioeconomic status. Additionally, being male, having a foreign-born, or younger mother more likely increases dropout, as described long ago (85). An earlier systematic review investigating neurodevelopmental impairment (including 24 cohorts of extremely preterm children— <1,000 g or born before 28 weeks of gestation) suggested that those lost to follow-up tended to be healthier than the participants (86), which might result from parents perception about their children being healthy and not needing to be followed. In this systematic review, there was a lack of uniformity in the impact of infants or children health-related characteristics and probability of non-participation. Few individual studies (85, 87) had earlier pointed the same paradox of extremes of better or worse health being more common in non-participants but more detailed information about parents and children's perception of the relevance to participate in long-term research seems needed.

Birth cohorts offer a unique opportunity to monitor early-life factors associated with variation in growth and development (88), becoming a valuable instrument to investigate the long-term effects of prematurity. However, these types of studies pose methodological challenges due to attrition, especially because it is not possible to replace those lost to follow-up since eligibility is linked to date of birth (82). The financial and time-consuming nature of a cohort study highlights the relevance of optimizing factors that contribute to quality and validity of the collected data. A review conducted on participation bias using three high-impact journals of epidemiology showed that non-response bias is often ignored or dismissed as negligible (18), which acquiesces with the lack of detailed information identified in the present systematic review.

When it comes to health policies translation, the present study raises concern, as it demonstrated that very few publications provided “enough information” on retention features in birth cohorts of very preterm infants, and this lack of information may blur the interpretation of the evidence and its usefulness. Even after the publication of the STROBE statement, the “completeness” of assessment did not increase in published work, contrary to expectations. By following STROBE recommendations, authors should ensure that retention features are reported with enough detail to allow readers to assess the validity of results. We stress that authors shall report the absolute numbers of participants: (a) potentially eligible, (b) examined for eligibility, (c) confirmed eligible, (d) included in the study, (e) completing follow-up, and (f) analyzed. Such detailed description will add value to any study by increasing its transparency and comparability, since retention might be inappropriately inflated if authors exclude individuals who have previously withdrawn, moved, or cannot be located.

This systematic review engaged in a comprehensive search without language or year of publication restrictions. Nonetheless, we screened publications initially based on the information provided in titles and abstracts; thus, we may have missed some relevant publications because they may only address retention features and attrition in the full text. Our inclusion criteria encompasses participants based on birth weight and gestational age, with no restriction regarding sample size or year of recruitment, which introduced heterogeneity between studies. However, restricting these criteria would have resulted in a limitation of the number of relevant publications included in this review. Finally, a paucity of data reported by the included publications and heterogeneity of birth cohort studies hampered a meta-analysis, occasioning a more descriptive profile of the present systematic review.

## Conclusion

Conclusions were limited by the lack of detailed information on retention features, unexpected after 10 years of the release of STROBE statement and the evidence of threats to internal and external validity associated with attrition. Our results stressed a relevant shortcoming hindering the use of evidence derived from birth cohort studies of very preterm infants for clinical and public health purpose. We recommend a clear reporting of retention features through the consistent use of present STROBE statement or any newly revised version that further emphasizes the importance of detailed quantitative and qualitative information on retention. Besides, a culture of awareness, improvement, and transparency on reporting retention features should be continuously fostered by researchers conducting longitudinal studies.

## Data Availability Statement

The datasets generated for this study will not be made publicly available, a systematic review includes a methodology that can be followed by any reader to check the results.

## Author Contributions

RT developed the search strategy, carried out study selection, data extraction, evidence synthesis, interpretation of data, and writing of initial manuscript and subsequent revisions. AQ, AF, and EL carried out study selection, data extraction, contribute to the interpretation of data, and reviewed the manuscript. AS contributed to the development of the search strategy and interpretation of the data, verified study inclusion decisions, and reviewed the manuscript. CM analyzed the data, provided statistical advice, and reviewed the manuscript. HB conceived of and designed the study, performed the final data analysis, and participated in the drafting, review, and revision of the manuscript. All authors contributed to the article and approved the submitted version.

## Conflict of Interest

The authors declare that the research was conducted in the absence of any commercial or financial relationships that could be construed as a potential conflict of interest.

## References

[1] Seaton SE King S Manktelow BN Draper ES Field DJ. Babies born at the threshold of viability: changes in survival and workload over 20 years. Arch Dis Child Fetal Neonatal Ed. (2013) 98:F15–20. 10.1136/fetalneonatal-2011-301572 22516474 PMC3479086

[2] Draper ES Zeitlin J Fenton AC Weber T Gerrits J Martens G. Investigating the variations in survival rates for very preterm infants in 10 European regions: the MOSAIC birth cohort. Arch Dis Child Fetal Neonatal Ed. (2009) 94:F158–63. 10.1136/adc.2008.141531 18805823

[3] Ancel P-Y Goffinet F Kuhn P Langer B Matis J Hernandorena X. Survival and morbidity of preterm children born at 22 through 34 weeks' gestation in France in 2011: results of the EPIPAGE-2 cohort study. JAMA Pediatr. (2015) 169:230–8. 10.1001/jamapediatrics.2014.3351 25621457

[4] Saigal S Doyle LW. An overview of mortality and sequelae of preterm birth from infancy to adulthood. Lancet. (2008) 371:261–9. 10.1016/S0140-6736(08)60136-1 18207020

[5] Allotey J Zamora J Cheong-See F Kalidindi M Arroyo-Manzano D Asztalos E. Cognitive, motor, behavioural and academic performances of children born preterm: a meta-analysis and systematic review involving 64 061 children. BJOG. (2018) 125:16–25. 10.1111/1471-0528.14832 29024294

[6] Sipola-Leppänen M Vääräsmäki M Tikanmäki M Matinolli H-M Miettola S Hovi P. Cardiometabolic risk factors in young adults who were born preterm. Am J Epidemiol. (2015) 181:861–73. 10.1093/aje/kwu443 25947956 PMC4445394

[7] Treyvaud K. Parent and family outcomes following very preterm or very low birth weight birth: a review. Semin Fetal Neonatal Med. (2014) 19:131–5. 10.1016/j.siny.2013.10.008 24252709

[8] Taylor HG Klein N Minich NM Hack M. Long-term family outcomes for children with very low birth weights. Arch Pediatr Adolesc Med. (2001) 155:155–61. 10.1001/archpedi.155.2.155 11177090

[9] Blencowe H Cousens S Oestergaard MZ Chou D Moller A-B Narwal R. National, regional, and worldwide estimates of preterm birth rates in the year 2010 with time trends since 1990 for selected countries: a systematic analysis and implications. Lancet. (2012) 379:2162–72. 10.1016/S0140-6736(12)60820-4 22682464

[10] Given BA Keilman LJ Collins C Given CW. Strategies to minimize attribution in longitudinal studies. Nurs Res. (1990) 39:184–6. 10.1097/00006199-199005000-00018 2342908

[11] Moore T Hennessy EM Myles J Johnson SJ Draper ES Costeloe KL. Neurological and developmental outcome in extremely preterm children born in England in 1995 and 2006: the EPICure studies. BMJ. (2012) 345:e7961. 10.1136/bmj.e7961 23212880 PMC3514471

[12] Wolke D Waylen A Samara M Steer C Goodman R Ford T. Selective drop-out in longitudinal studies and non-biased prediction of behaviour disorders. Br J Psychiatry. (2009) 195:249–56. 10.1192/bjp.bp.108.053751 19721116 PMC2802508

[13] Kristman VL Manno M Côté P. Methods to account for attrition in longitudinal data: do they work? A simulation study. Eur J Epidemiol. (2005) 20:657–62. 10.1007/s10654-005-7919-7 16151878

[14] Touloumi G Pocock SJ Babiker AG Darbyshire JH. Impact of missing data due to selective dropouts in cohort studies and clinical trials. Epidemiology. (2002):347–55. 10.1097/00001648-200205000-00017 11964938

[15] Booker CL Harding S Benzeval M. A systematic review of the effect of retention methods in population-based cohort studies. BMC Public Health. (2011) 11:249. 10.1186/1471-2458-11-249 21504610 PMC3103452

[16] Hunt JR White E. Retaining and tracking cohort study members. Epidemiol Rev. (1998) 20:57–70. 10.1093/oxfordjournals.epirev.a017972 9762509

[17] Marcellus L. Are we missing anything? Pursuing research on attrition. Can J Nurs Res Arch. (2004) 36:82–98. Available online at: https://cjnr.archive.mcgill.ca/article/view/1901 15551664

[18] Keeble C Barber S Law GR Baxter PD. Participation bias assessment in three high-impact journals. Sage Open. (2013) 3:1–5. 10.1177/2158244013511260

[19] Moher D Liberati A Tetzlaff J Altman DG Group P. Preferred reporting items for systematic reviews and meta-analyses: the PRISMA statement. PLoS Med. (2009) 6:e1000097. 10.1371/journal.pmed.1000097 19621072 PMC2707599

[20] Cohen J. Weighted kappa: nominal scale agreement provision for scaled disagreement or partial credit. Psychol Bull. (1968) 70:213. 10.1037/h0026256 19673146

[21] Morton LM Cahill J Hartge P. Reporting participation in epidemiologic studies: a survey of practice. Am J Epidemiol. (2005) 163:197–203. 10.1093/aje/kwj036 16339049

[22] Von Elm E Altman DG Egger M Pocock SJ Gøtzsche PC Vandenbroucke JP. The Strengthening the Reporting of Observational Studies in Epidemiology (STROBE) statement: guidelines for reporting observational studies. Ann Intern Med. (2007) 147:573–7. 10.7326/0003-4819-147-8-200710160-00010 17938396

[23] Cleveland WS Grosse E. Computational methods for local regression. Stat Comput. (1991) 1:47–62. 10.1007/BF01890836

[24] Cleveland WS. Robust locally weighted regression and smoothing scatterplots. J Am Stat Assoc. (1979) 74:829–36. 10.1080/01621459.1979.10481038

[25] Cleveland WS Devlin SJ Grosse E. Regression by local fitting: methods, properties, and computational algorithms. J Econom. (1988) 37:87–114. 10.1016/0304-4076(88)90077-2

[26] Pierrat V Marchand-Martin L Arnaud C Kaminski M Resche-Rigon M Lebeaux C. Neurodevelopmental outcome at 2 years for preterm children born at 22 to 34 weeks' gestation in France in 2011: EPIPAGE-2 cohort study. BMJ. (2017) 358:j3448. 10.1136/bmj.j3448 28814566 PMC5558213

[27] Brévaut-Malaty V Busuttil M Einaudi M-A Monnier A-S D'Ercole C Gire C. Longitudinal follow-up of a cohort of 350 singleton infants born at less than 32 weeks of amenorrhea: neurocognitive screening, academic outcome, and perinatal factors. Eur J Obstetr Gynecol Reprod Biol. (2010) 150:13–8. 10.1016/j.ejogrb.2010.01.001 20106582

[28] Guellec I Lapillonne A Renolleau S Charlaluk M-L Roze J-C Marret S. Neurologic outcomes at school age in very preterm infants born with severe or mild growth restriction. Pediatrics. (2011) 127:e883-e91. 10.1542/peds.2010-2442 21382951

[29] Ancel P-Y Livinec F Larroque B Marret S Arnaud C Pierrat V. Cerebral palsy among very preterm children in relation to gestational age and neonatal ultrasound abnormalities: the EPIPAGE cohort study. Pediatrics. (2006) 117:828–35. 10.1542/peds.2005-0091 16510664

[30] Larroque B Ancel P-Y Marret S Marchand L André M Arnaud C. Neurodevelopmental disabilities and special care of 5-year-old children born before 33 weeks of gestation (the EPIPAGE study): a longitudinal cohort study. Lancet. (2008) 371:813–20. 10.1016/S0140-6736(08)60380-3 18328928

[31] Farooqi A Hägglöf B Serenius F. Behaviours related to executive functions and learning skills at 11 years of age after extremely preterm birth: a Swedish national prospective follow-up study. Acta Paediatr. (2013) 102:625–34. 10.1111/apa.12219 23458380

[32] Hellgren KM Tornqvist K Jakobsson PG Lundgren P Carlsson B Källén K. Ophthalmologic outcome of extremely preterm infants at 6.5 years of age: extremely preterm infants in sweden study (EXPRESS). JAMA Ophthalmol. (2016) 134:555–62. 10.1001/jamaophthalmol.2016.0391 27010699

[33] Serenius F Källén K Blennow M Ewald U Fellman V Holmström G. Neurodevelopmental outcome in extremely preterm infants at 2.5 years after active perinatal care in Sweden. JAMA. (2013) 309:1810–20. 10.1001/jama.2013.3786 23632725

[34] Finnström O Olausson PO Sedin G Serenius F Svenningsen N Thiringer K. Neurosensory outcome and growth at three years in extremely low birthweight infants: follow-up results from the Swedish national prospective study. Acta Paediatr. (1998) 87:1055–60. 10.1111/j.1651-2227.1998.tb01413.x 9825972

[35] Finnström O Gäddlin P-O Leijon I Samuelsson S Wadsby M. Very-low-birth-weight children at school age: academic achievement, behavior and self-esteem and relation to risk factors. J Mat Fetal Neonatal Med. (2003) 14:75–84. 10.1080/jmf.14.2.75.84 14629086

[36] Elgen SK Sommerfelt K Leversen KT Markestad T. Minor neurodevelopmental impairments are associated with increased occurrence of ADHD symptoms in children born extremely preterm. Eur Child Adolesc Psychiatry. (2015) 24:463–70. 10.1007/s00787-014-0597-9 25304291

[37] Husby IM Skranes J Olsen A Brubakk A-M Evensen KAI. Motor skills at 23 years of age in young adults born preterm with very low birth weight. Early Hum Dev. (2013) 89:747–54. 10.1016/j.earlhumdev.2013.05.009 23810435

[38] Løhaugen GC Gramstad A Evensen KAI Martinussen M Lindqvist S Indredavik M. Cognitive profile in young adults born preterm at very low birthweight. Dev Med Child Neurol. (2010) 52:1133–8. 10.1111/j.1469-8749.2010.03743.x 21175467

[39] Vederhus BJ Markestad T Eide GE Graue M Halvorsen T. Health related quality of life after extremely preterm birth: a matched controlled cohort study. Health Qual Life Outcomes. (2010) 8:53. 10.1186/1477-7525-8-53 20492724 PMC2894784

[40] Evensen KAI Lindqvist S Indredavik MS Skranes J Brubakk A-M Vik T. Do visual impairments affect risk of motor problems in preterm and term low birth weight adolescents? Eur J Paediatr Neurol. (2009) 13:47–56. 10.1016/j.ejpn.2008.02.009 18430596

[41] Hille E Den Ouden A Stuifbergen M Verrips G Vogels A Brand R. Is attrition bias a problem in neonatal follow-up? Early Hum Dev. (2005) 81:901–8. 10.1016/j.earlhumdev.2005.07.006 16150560

[42] Hille ET Weisglas-Kuperus N Van Goudoever J Jacobusse GW Ens-Dokkum MH de Groot L. Functional outcomes and participation in young adulthood for very preterm and very low birth weight infants: the Dutch Project on Preterm and Small for Gestational Age Infants at 19 years of age. Pediatrics. (2007) 120:e587-95. 10.1542/peds.2006-2407 17766499

[43] Stoelhorst GM Rijken M Martens SE van Zwieten PH Feenstra J Zwinderman AH. Developmental outcome at 18 and 24 months of age in very preterm children: a cohort study from 1996 to 1997. Early Hum Dev. (2003) 72:83–95. 10.1016/S0378-3782(03)00011-2 12782421

[44] Johnson S Hollis C Kochhar P Hennessy E Wolke D Marlow N. Psychiatric disorders in extremely preterm children: longitudinal finding at age 11 years in the EPICure study. J Am Acad Child Adolesc Psychiatry. (2010) 49:e7961. 10.1016/j.jaac.2010.02.002 20431465

[45] Wood NS Marlow N Costeloe K Gibson AT Wilkinson AR. Neurologic and developmental disability after extremely preterm birth. New Engl J Med. (2000) 343:378–84. 10.1056/NEJM200008103430601 10933736

[46] Dinesen S Greisen G. Quality of life in young adults with very low birth weight. Arch Dis Child Fetal Neonatal Ed. (2001) 85:F165–9. 10.1136/fn.85.3.F165 11668156 PMC1721339

[47] Hansen BM Hoff B Greisen G Mortensen E. Early nasal continuous positive airway pressure in a cohort of the smallest infants in Denmark: neurodevelopmental outcome at five years of age. Acta Paediatr. (2004) 93:190–5. 10.1111/j.1651-2227.2004.tb00704.x 15046272

[48] Jaekel J Wolke D Bartmann P. Poor attention rather than hyperactivity/impulsivity predicts academic achievement in very preterm and full-term adolescents. Psychol Med. (2013) 43:183–96. 10.1017/S0033291712001031 22608065

[49] Voss W Hobbiebrunken E Ungermann U Wagner M Damm G. The development of extremely premature infants: results from the lower saxony longitudinal study of prematurity (Niedersächsisches Frühgeborenen-Nachuntersuchungsprojekt). Deutsches Ärzteblatt Int. (2016) 113:871. 10.3238/arztebl.2016.0871 PMC5282475 28130919

[50] Bertino E Coscia A Mombrò M Boni L Rossetti G Fabris C. Postnatal weight increase and growth velocity of very low birthweight infants. Arch Dis Child Fetal Neonatal Ed. (2006) 91:F349–56. 10.1136/adc.2005.090993 16638781 PMC2672838

[51] Cuttini M Caravale B Carnielli V Chiandotto V Contoli B Corchia C. A two-year follow-up study of very preterm infants in Italy: aims and study design. Paediatr Child Health. (2009) 19:S145–52. 10.1016/j.paed.2009.08.027

[52] Räikkönen K Pesonen A-K Heinonen K Kajantie E Hovi P Järvenpää A-L. Depression in young adults with very low birth weight: the Helsinki study of very low-birth-weight adults. Arch Gen Psychiatry. (2008) 65:290–6. 10.1001/archgenpsychiatry.2007.40 18316675

[53] Mikkola K Ritari N Tommiska V Salokorpi T Lehtonen L Tammela O. Neurodevelopmental outcome at 5 years of age of a national cohort of extremely low birth weight infants who were born in 1996–1997. Pediatrics. (2005) 116:1391–400. 10.1542/peds.2005-0171 16322163

[54] Hall A McLeod A Counsell C Thomson L Mutch L. School attainment, cognitive ability and motor function in a total scottish verylow-birthweight population at eight years: a controlled study. Dev Med Child Neurol. (1995) 37:1037–50. 10.1111/j.1469-8749.1995.tb11965.x 8566462

[55] Kiechl-Kohlendorfer U Ralser E Peglow UP Reiter G Trawöger R. Adverse neurodevelopmental outcome in preterm infants: risk factor profiles for different gestational ages. Acta Paediatr. (2009) 98:792–6. 10.1111/j.1651-2227.2009.01219.x 19191762

[56] De Groote I Vanhaesebrouck P Bruneel E Dom L Durein I Hasaerts D. Outcome at 3 years of age in a population-based cohort of extremely preterm infants. Obstetr Gynecol. (2007) 110:855–64. 10.1097/01.AOG.0000284447.43442.55 17906020

[57] Saigal S Rosenbaum P Stoskopf B Hoult L Furlong W Feeny D. Comprehensive assessment of the health status of extremely low birth weight children at eight years of age: comparison with a reference group. J Pediatr. (1994) 125:411–7. 10.1016/S0022-3476(05)83288-3 8071751

[58] Saigal S Stoskopf B Streiner D Boyle M Pinelli J Paneth N. Transition of extremely low-birth-weight infants from adolescence to young adulthood: comparison with normal birth-weight controls. JAMA. (2006) 295:667–75. 10.1001/jama.295.6.667 16467235

[59] Rogers M Fay TB Whitfield MF Tomlinson J Grunau RE. Aerobic capacity, strength, flexibility, and activity level in unimpaired extremely low birth weight (≤800 g) survivors at 17 years of age compared with term-born control subjects. Pediatrics. (2005) 116:e58–65. 10.1542/peds.2004-1603 15997047

[60] Saigal S Stoskopf BL Streiner DL Burrows E. Physical growth and current health status of infants who were of extremely low birth weight and controls at adolescence. Pediatrics. (2001) 108:407–15. 10.1542/peds.108.2.407 11483807

[61] Saigal S Day KL Van Lieshout RJ Schmidt LA Morrison KM Boyle MH. Health, wealth, social integration, and sexuality of extremely low-birth-weight prematurely born adults in the fourth decade of life. JAMA Pediatr. (2016) 170:678–86. 10.1001/jamapediatrics.2016.0289 27213291

[62] McManus BM Robert S Albanese A Sadek-Badawi M Palta M. Predictors of receiving therapy among very low birth weight 2-year olds eligible for Part C early intervention in Wisconsin. BMC Pediatr. (2013) 13:106. 10.1186/1471-2431-13-106 23845161 PMC3718652

[63] Gross SJ Mettelman BB Dye TD Slagle TA. Impact of family structure and stability on academic outcome in preterm children at 10 years of age. J Pediatr. (2001) 138:169–75. 10.1067/mpd.2001.111945 11174612

[64] Anderson PJ Doyle LW. Executive functioning in school-aged children who were born very preterm or with extremely low birth weight in the 1990s. Pediatrics. (2004) 114:50–7. 10.1542/peds.114.1.50 15231907

[65] Anderson PJ De Luca CR Hutchinson E Roberts G Doyle LW. Underestimation of developmental delay by the new Bayley-III Scale. Arch Pediatr Adolesc Med. (2010) 164:352–6. 10.1001/archpediatrics.2010.20 20368488

[66] Doyle LW Cheong JL Burnett A Roberts G Lee KJ Anderson PJ. Biological and social influences on outcomes of extreme-preterm/low-birth weight adolescents. Pediatrics. (2015) 136:e1513–20. 10.1542/peds.2015-2006 26553187

[67] Danks M Burns YR Gibbons K Gray PH O'callaghan MJ Poulsen L. Fitness limitations in non-disabled extremely low birthweight adolescents. J Paediatr Child Health. (2013) 49:548–53. 10.1111/jpc.12281 23751052

[68] Hutchinson EA De Luca CR Doyle LW Roberts G Anderson PJ Group VICS. School-age outcomes of extremely preterm or extremely low birth weight children. Pediatrics. (2013) 131:e1053-61. 10.1542/peds.2012-2311 23509167

[69] Mills IS Doyle LW Cheong JL Roberts G. Rates of early intervention services in children born extremely preterm/extremely low birthweight. J Paediatr Child Health. (2018) 54:74–9. 10.1111/jpc.13668 28800210

[70] Ullman H Spencer-Smith M Thompson DK Doyle LW Inder TE Anderson PJ. Neonatal MRI is associated with future cognition and academic achievement in preterm children. Brain. (2015) 138:3251–62. 10.1093/brain/awv244 26329284 PMC4731414

[71] Kitchen W Ford G Orgill A Rickards A Astbury J Lissenden J. Outcome in infants of birth weight 500 to 999 g: a continuing regional study of 5-year-old survivors. J Pediatr. (1987) 111:761–6. 10.1016/S0022-3476(87)80264-0 2959764

[72] Groupyy VICS. Eight-year outcome in infants with birth weight of 500 to 999 grams: continuing regional study of 1979 and 1980 births. J Pediatr. (1991) 118:761–7. 10.1016/S0022-3476(05)80044-7 2019933

[73] Roberts G Lim J Doyle LW Anderson PJ. High rates of school readiness difficulties at 5 years of age in very preterm infants compared with term controls. J Dev Behav Pediatr. (2011) 32:117–24. 10.1097/DBP.0b013e318206d5c9 21169858

[74] Roberts G Howard K Spittle AJ Brown NC Anderson PJ Doyle LW. Rates of early intervention services in very preterm children with developmental disabilities at age 2 years. J Paediatr Child Health. (2008) 44:276–80. 10.1111/j.1440-1754.2007.01251.x 17999667

[75] Molloy CS Wilson-Ching M Anderson VA Roberts G Anderson PJ Doyle LW. Visual processing in adolescents born extremely low birth weight and/or extremely preterm. Pediatrics. (2013) 132:e704–12. 10.1542/peds.2013-0040 23918899

[76] Murray AL Scratch SE Thompson DK Inder TE Doyle LW Anderson JF. Neonatal brain pathology predicts adverse attention and processing speed outcomes in very preterm and/or very low birth weight children. Neuropsychology. (2014) 28:552. 10.1037/neu0000071 24708047 PMC4106799

[77] Doyle L Casalaz D. Outcome at 14 years of extremely low birthweight infants: a regional study. Arch Dis Child Fetal Neonatal Ed. (2001) 85:F159–64. 10.1136/fn.85.3.F159 11668155 PMC1721322

[78] Victorian Infant Collaborative Study Group. Postnatal corticosteroids and sensorineural outcome at 5 years of age. J Paediatr Child Health. (2000) 36:256–61. 10.1046/j.1440-1754.2000.00493.x 10849228

[79] Hayes M Kelly E Knoches A McDougall P Rickards A Watkins A. Neurosensory outcome at 5 years and extremely low birthweight. Arch Dis Child. (1995) 73:F143–6. 10.1136/fn.73.3.F143 8535869 PMC2528475

[80] Horwood LJ Mogridge N Darlow BA. Cognitive, educational, and behavioural outcomes at 7 to 8 years in a national very low birthweight cohort. Arch Dis Child Fetal Neonatal Ed. (1998) 79:F12–20. 10.1136/fn.79.1.F12 9797619 PMC1720817

[81] Tu Y-F Wang L-W Wang S-T Yeh T-F Huang C-C. Postnatal steroids and febrile seizure susceptibility in preterm children. Pediatrics. (2016) 137:e20153404. 10.1542/peds.2015-3404 27012746

[82] Golding J Birmingham K. Enrollment and response rates in a longitudinal birth cohort. Paediatr Perinat Epidemiol. (2009) 23(Suppl. 1):73–85. 10.1111/j.1365-3016.2008.01001.x 19490447

[83] Davis LL Broome ME Cox RP. Maximizing retention in community-based clinical trials. J Nurs Scholarsh. (2002) 34:47–53. 10.1111/j.1547-5069.2002.00047.x 11901967

[84] Kristman V Manno M Côté P. Loss to follow-up in cohort studies: how much is too much? Eur J Epidemiol. (2004) 19:751–60. 10.1023/B:EJEP.0000036568.02655.f8 15469032

[85] Wolke D Söhne B Ohrt B Riegel K. Follow-up of preterm children: important to document dropouts. Lancet. (1995) 345:447. 10.1016/S0140-6736(95)90425-5 7531802

[86] Guillén U DeMauro S Ma L Zupancic J Roberts R Schmidt B. Relationship between attrition and neurodevelopmental impairment rates in extremely preterm infants at 18 to 24 months: a systematic review. Arch Pediatr Adolesc Med. (2012) 166:178–84. 10.1001/archpediatrics.2011.616 22312176

[87] McCormick MC Baker J Brooks-Gunn J Turner J Workman-Daniels K Peckham GJ. Cohort reconstruction: which infants can be restudied at school age? Paediatr Perinatal Epidemiol. (1991) 5:410–22. 10.1111/j.1365-3016.1991.tb00727.x 1754500

[88] Larsen PS Kamper-Jørgensen M Adamson A Barros H Bonde JP Brescianini S. Pregnancy and birth cohort resources in europe: a large opportunity for aetiological child health research. Paediatr Perinat Epidemiol. (2013) 27:393–414. 10.1111/ppe.12060 23772942

